# Management of cardiac fibrosis in diabetic rats; the role of peroxisome proliferator activated receptor gamma (PPAR-gamma) and calcium channel blockers (CCBs)

**DOI:** 10.1186/1758-5996-3-4

**Published:** 2011-03-30

**Authors:** Hoda E Mohamad, Mervat E Askar, Mohamed M Hafez

**Affiliations:** 1 Department of Biochemistry, Faculty of Pharmacy, Zagazig University, Zagazig, Egypt; 2 Department of Biochemistry, Faculty of Pharmacy, October for Modern Science and Arts University (MSA), Egypt

## Abstract

**Background:**

Diabetes mellitus (DM) and hypertension (HTN) are accused of being responsible for the development of the cardiac fibrosis due to severe cardiomyopathy.

**Methods:**

Blood glucose (BG) test was carried out, lipid concentrations, tumor necrosis factor alpha (TNF-α), transforming growth factor beta (TGF-β), matrix metalloproteinase (MMP-2), collagen-I and collagen-III were measured in male *Albino *rats weighing 179-219 g. The rats were divided into five groups, kept on either control diet or high fat diet (HFD), and simultaneously treated with rosiglitazone (PPAR-gamma) only for one group with 3 mg/kg/day via oral route for 30 days, and with rosiglitazone and felodipine combination for another group with 3 mg/kg/day and 5 mg/kg/day, respectively via oral route for 30 days.

**Results:**

Diabetic hypertensive (DH) rats which fed on a HFD, injected with streptozotocin (STZ) (i.p.) and obstruction for its right kidney was occurred develop hyperglycemia, hypertension, cardiac fibrosis, hypertriglyceridemia, hypercholesterolemia, increased TNF-α, increased TGF-β, decreased MMP-2, increased collagen-I and increased collagen-III, when compared to rats fed on control diet. Treating the DH rats with rosiglitazone only causes a significant decrease for BG levels by 52.79%, triglycerides (TGs) by 24.05%, total cholesterol (T-Chol) by 30.23%, low density lipoprotein cholesterol (LDL-C) by 40.53%, TNF-α by 20.81%, TGF-β by 46.54%, collagen-I by 48.11% and collagen-III by 53.85% but causes a significant increase for MMP-2 by 272.73%. Moreover, Treating the DH rats with rosiglitazone and felodipine combination causes a significant decrease for BG levels by 61.08%, blood pressure (BP) by 16.78%, TGs by 23.80%, T-Chol by 33.27%, LDL-C by 45.18%, TNF-α by 22.82%, TGF-β by 49.31%, collagen-I by 64.15% and collagen-III by 53.85% but causes a significant increase for MMP-2 by 290.91%. Rosiglitazone alone failed to decrease the BP in DH rats in the current dosage and duration.

**Conclusion:**

Our results indicate that the co-existence of diabetes and hypertension could induce cardiomyopathy which could further result in cardiac fibrosis, and that combination treatment with rosiglitazone and felodipine has a great protective role against the metabolic abnormalities, meanwhile, the treatment with rosiglitazone alone has a protective role with a minimal effect against these abnormalities and has no effect on decreasing BP in these cases which may lead to coronary artery diseases (CADs) in future.

## Introduction

Diabetic cardiomyopathy, the leading cause of death in diabetic patients, is characterized by both systolic and diastolic dysfunction, due to reduced contractility, prolonged relaxation, and decreased compliance [1,2]. The development of diabetic cardiomyopathy is multifactorial. Putative mechanisms include metabolic disturbances, small vessel disease, autonomic dysfunction, insulin resistance, and myocardial fibrosis [3,4]. Recently interstitial fibrosis has been regarded as an important pathogenic factor of the heart's impaired functional integrity [5]; however, altered substrate supply and use by cardiac myocytes could be the primary injury in the pathogenesis of this specific heart muscle disease. Structural and functional impairment of myocytes, which occurs already in the first week of diabetes, precedes cardiac fibrosis and thus remains the key step in impairing contractile performance, finally inducing heart failure [6]. The diagnosis of a diabetic cardiomyopathy is made in patients in whom no other known etiological factors, such as coronary artery disease, or hypertension, are present [7].

Diabetes and high blood pressure are closely related diseases. They occur together so frequently that they are officially considered to be "comorbidities" (diseases likely to be present in the same patient). Unfortunately, diabetes makes high blood pressure more difficult to treat, and high blood pressure makes diabetes even more dangerous [8].

Streptozotocin (STZ) is a naturally occurring, broad-spectrum antibiotic [9]. Induction of experimental diabetes in the rat using STZ is very convenient and simple to use but it is cyto-toxic chemical that is particularly toxic to the pancreatic, insulin-producing beta cells in mammals. STZ injection leads to the degeneration of the Langerhans islets beta cells [10].

One class of insulin sensitizers of the invention is Peroxisome Proliferator-Activated Receptors (PPAR) modulators, and in particular PPAR-gamma modulators, e.g., PPAR-gamma agonists. PPARs are members of the nuclear hormone receptor superfamily of ligand-activated transcription factors that are related to retinoid, steroid and thyroid hormone receptors. PPAR modulators include the PPAR-alpha, PPAR-delta (also called PPAR-beta), and PPAR-gamma agonists. Especially useful are the thiazolidinediones (TZDs), which are exogenous substances, used for clinicians in diabetic patients and not expressed in any tissue. TZDs act upon PPAR-gamma. PPAR-gamma are expressed in many tissues especially the heart [11]. They were developed in the 70's and 80's by screening newly synthesized compounds for their ability to lower blood glucose in diabetic rodents. Three molecules from this class, troglitazone, rosiglitazone, and pioglitazone, were ultimately approved for the treatment of patients with type II diabetes [12].

Although these compounds were developed without an understanding of their molecular mechanism of action, by the early 90's evidence began to accumulate linking the thiazolidinediones to the nuclear receptor PPAR-gamma. It was ultimately demonstrated that these molecules were high affinity ligands of PPAR-gamma and that they increased transcriptional activity of the receptor. Ligands of PPAR-gamma including certain thiazolidinediones, reduce myocardial tissue injury and infarct size [13-15]. TZDs PPAR-gamma activators are used clinically to improve insulin sensitivity and glycemic control in patients with Type II diabetes. PPAR-gamma is predominantly expressed in adipose tissue, but is also expressed in myocardium [16]. The function of PPAR-gamma in the heart suggests that activation of myocardial PPAR-gamma may have metabolic and anti-inflammatory effects. PPAR-gamma activation has been shown to increase myocardial expression of glucose transporters, promote carbohydrate substrate utilization in cardiac myocytes and intact hearts [17,18], and attenuate pro-inflammatory cytokine expression in activated cardiomyocytes [19], experimental myocarditis [20] or chronic LV failure due to myocardial infarction [21]. Moreover, most studies have shown that TZDs PPAR-gamma activators reduce myocardial infarct size and enhance recovery of contractile function in intact rat hearts after ischemia and reperfusion. These findings are derived from studies in both normal and insulin resistant models [17,18,22,23].

Metabolic syndrome is associated with accelerated macrovascular and microvascular coronary disease, cardiomyopathy, and elevated inflammatory status [24-27]. Felodipine (which used as calcium channel blocker) administration can attenuate the inflammatory and fibrosis process. As felodipine is used for treating high blood pressure. This medication helps to relax the blood vessels, which improves blood flow and makes it easier for the heart to pump blood. It has a direct beneficial effect by inhibiting the nuclear translocation and DNA-binding activity of nuclear factor-κB, which plays a central role in this process by regulating the expression of several proinflammatory genes [28].

TNF-α is an acute phase reactive protein and a basic medium of immunological regulation, and it is also an inflammatory cytokine with pleiotropic biological effects. In the circulatory system, TNF-α is responsible for many myocardial diseases such as acute coronary syndrome [29], Acute myocardial infarction (AMI) [30,31], myocarditis [32], dilated cardiomyopathy and congestive heart failure (CHF) [33,34].

TGF-β1 facilitates fibrous tissue formation, upregulates collagen expression by stimulating extracellular matrix synthesis, and mediates perivascular and myocardial fibrosis. The increased gene expression of extracellular matrix (ECM) components, such as collagen, and of TGF-ß1 causes cardiac fibrosis and promotes cardiac stiffness, leading to diastolic dysfunction [35].

Myocardial stunning was attenuated by inhibition of MMP-2 activity [36,37]. In the setting of heart failure, MMP-2 has been implicated in myocardial fibrosis, increased myocardial stiffness and impaired contractility of hearts from spontaneously hypertensive rats [38].

Cardiac fibroblasts are the most abundant cell type of the myocardium and produce various proteins found in ECM, including the major fibrillar collagens type I and III, which comprise the bulk of the ECM, as well as collagenases, fibronectin, and vitronectin [39].

Calcium channel blockers (CCBs) are widely used to treat cardiovascular diseases [40].

In this study, we investigate the role of CCBs (felodipine) and PPAR-gamma modulators on blood glucose level, blood pressure, lipid profile, TNF-α, TGF-β1, MMP-2, collagen-I and collagen-III in DH rats.

## Materials and methods

### Chemicals

Glucose (Spectrum Diagnostics, Cairo, Egypt). STZ (Sigma chemical Company, Saint Louis, MO, USA).

### Diet

The diet for forty rats were fed a high-fat diet consists of 22.5% hydrogenated vegetable oil, 22.5% milk powder, 51.5% soybean ground, 2% corn starch, 1% sucrose and 0.5% vitamins and minerals.

### Animals

Fifty male *Albino *rats were used for the present study after being procured from the animal house of El-Nile Company for Pharmaceutical Products (Cairo, Egypt). The animals were acclimatized for two weeks in the animal house of Zagazig University before dietary manipulation. After two weeks of high-fat diet, induction of diabetes was occurred, as rats were overnight fasted and injected intra-peritoneal (i.p.) with 35 mg/kg **STZ (Sigma chemical Company, Saint Louis, MO, USA) **freshly prepared in cold 0.1 mol citrate buffer (pH 4.5) [41]. One week after the injection of STZ hyperglycemic state was first tested by detection of glucosuria using urine glucose strips. Rats with two successive blood glucose levels more than 300 mg/dl were used in this study. Thus streptozotocin injury was occurred.

Then, induction of hypertension was occurred, as thirty diabetic rats were anesthetized injection of sodium thiopental (12 mg/100 gm) [42]. After anesthesia, the animal was fixed in supine position on the operating table and the abdominal skin was shaved and sterilized with 70% ethyl alcohol. Then, a midline incision was made, and the right renal artery was clamped for 45 minutes using a non-traumatic vascular clamp (Bulldog clamp). Then, the edges of the abdominal incision were approximated to each other and covered by a piece of gauze soaked with warm isotonic saline (37°C) to prevent undue loss of body fluids. Reperfusion achieved after 15 minutes from removal of the vascular clamp on the right renal artery [43].

Two rats were housed per wire floored cage in an air-conditioned room (22 ± 2°C) with 12 h light/dark cycle and had free access to standard laboratory chow diet (El Nasr Co, Cairo, Egypt), and water *ad libitum*. The protocol of the current study was approved by the department of Biochemistry Council, Faculty of Pharmacy, Zagazig University, which has an ethical authority.

### Experimental design

Animals weighed 179-219 g at the time of dietary manipulation. They were randomly assigned into five groups of ten each, as given below:

i. Control group (C): Normal control healthy rats.

ii. Diabetic Control group (DC): Diabetic control rats, that took fat diet for two weeks then injected with STZ intraperitoneal (i.p.).

iii. Diabetic Hypertensive Control group (DHC): Type 2 DM hypertensive rats, that took fat diet for two weeks then injected with STZ i.p. Finally, obstruction for right kidney was done for all of them for induction of hypertension.

iv. DH rats + Rosiglitazone (R): Treated DH rats with PPAR-gamma (Rosiglitazone) with a dose of 3 mg/kg/day [44] orally only for one month.

v. DH rats + combination of Rosiglitazone and Felodipine (R+F): Treated DH rats with a combination of PPAR-gamma (Rosiglitazone) with a dose of 3 mg/kg/day orally and CCB (Felodipine) with a dose of 5 mg/kg/day [45] orally for one month.

The animals were maintained in their respective groups for 30 days. Fasting serum glucose level, blood pressure, lipid profile, TNF-α, TGF-β1, MMP-2, collagen-I and collagen-III of all animals were measured at the last day (day 30) of the experiment.

### Blood Sample collection

Blood samples were collected from retro-orbital plexus of the eye after 30 days from 12-h fasted rats into vacutainer clotted tubes, where sera were obtained by centrifugation within 30 min at 4000 rpm at 4°C for 10 min using Centurion centrifuge (K280R, UK), then divided into aliquots and kept at -80°C for further assay of lipid profile and TNF-α while serum glucose was determined immediately.

### Harvesting of left ventricle specimen

The left ventricle of heart was removed rapidly and cut into two equal halves by a scalpel. One half of it was rapidly placed in 10% neutral buffered formalin for immunostaining and the other half was rapidly frozen in liquid nitrogen, and stored at -72°C for real time polymerase chain reactions (RT-PCR) assessments for TGF-β1, MMP-2, collagen-I and collagen-III [46].

### Histological examination

1) The selected paraffin blocks for immunohistochemical staining were sectioned (4- μm thickness) and stained with hematoxylin and eosin (H&E) stain [47].

2) Masson trichrome stain [48]: The data were obtained using Leica Qwin 500 image analyzer computer system (England). The image analyzer consisted of a coloured video camera, coloured monitor, hard disc of IBM personal computer connected to the microscope, and controlled by Leica Qwin 500 software. The image analyzer was first calibrated automatically to convert the measument units (pixels) produced by the image analyzer program into actual micrometer units.

The area % of CT fibers was measured using an objective lens of magnification 10, i.e of a total magnification of 100. Ten fields were measured for each specimen. Using the colour detect, CT areas were masked by a blue binary colour. The area % was calculated in relation to a standard measuring frame of area 118476.6 micrometer square.

The data obtained were subjected to statistical analysis using t-student's test and ANOVA.

### Biochemical measurements

The concentration of serum glucose was measured by the enzymatic colorimetric GOD-POD procedure [49] using Diamond Diagnostic kit (Germany).

Serum TGs were estimated by GPO-POD enzymatic method [50] using a Biocon kit (India). T-Chol concentration was determined utilizing enzymatic colorimetric CHOD-PAP method [51] using Biocon kit (India). HDL-C was determined by the same method after the precipitation of very low density lipoprotein cholesterol (VLDL-C) and LDL-C [52], and finally, LDL-C was calculated by Using Friedewald's Formula [53]:

Serum TNF-α was assessed by the rat TNF- alpha enzyme-linked immunosorbent assay (ELISA) kit which is an in vitro enzyme-linked immunosorbent assay for the quantitative measurement of rat TNF- alpha cell lysate and tissue lysate [54].

For the detection of collagen-I, collagen-III, TGF-β and MMP-2 gene expression, RNA was extracted, reverse transcribed into cDNA and amplified by PCR. Total RNA was extracted from heart tissue using SV Total RNA Isolation System (Promega, Madison, WI, USA). The extracted RNA was reverse transcribed into cDNA using RT-PCR kit (Stratagene, USA). Then amplification of specific DNA sequences using two primers that hybridize to opposite strands and flank target DNA region. At the end of the amplification process, the DNA product was detected using agarose gel electrophoresis.

### Statistical analysis

The results were expressed as mean ± SD. To determine the statistical significance of laboratory findings, multiple comparisons were achieved using independent samples T- Test and ANOVA followed by Tukey test as post hoc test. The correlations between BG and TNF-α were tested by Pearson's coefficient (r). P-value ≤ 0.05 was considered statistically significant.

## Results

DC animals showed significant increase in their serum glucose levels by 278.88% in comparison with group C. Furthermore, DHC animals showed also significant increase in their serum glucose levels by 35.15% in comparison with DC animals group. However, group R and group R+F induced significant decrease in their serum glucose levels by (52.79% and 61.08% respectively) in comparison with DHC data (Table 1).

**Table 1:** Effect of STZ and obstruction for right kidney on fasting serum glucose and blood pressure

Groups					
	**Control**	**Diabetic Control**	**Diabetic Hypertensive Control**	**Rosiglitazone**	**Rosiglitazone+Felodipine**

Glucose (mg/dL)	98.00 ± 9.61	371.30 ± 39.09 ^	501.80 ± 54.44*	236.90 ± 47.98#	195.30 ± 47.35#

Blood Pressure (mmHg)	126.20 ± 4.78	158.40 ± 14.09^	195.50 ± 13.26*	189.90 ± 13.16	162.70 ± 16.73#a

Values are expressed as mean ± SD. Number of rats per group n = 10.

STZ, streptozotocin

^ Significant difference from normal control at P < 0.05.

* Significant difference from diabetic control at P < 0.05.

# Significant difference from diabetic hypertensive control at P < 0.05.

a Means of treated groups with different superscript are significantly different from each other at P < 0.05.

DC animals showed significant increase in their BP levels by 25.52% in comparison with group C. Furthermore, DHC animals showed also significant increase in their BP levels by 23.42% in comparison with DC animals group. But there is no significance between group R and DHC data in its BP levels. However, group R+F induced significant decrease in their BP levels by 16.78% in comparison with DHC data (Table 1).

DC animals showed significant increase in their TGs levels by 20.06% in comparison with group C. But, DHC animals showed no significance in their TAG levels in comparison with DC animals group. Meanwhile, there was nearly the same significant decrease between group R and group R+F in their TAG levels by (24.05% and 23.80% respectively) in comparison with DHC data (Table 2).

**Table 2:** Measurements of fasting serum lipids, TNF-α, TGF-β, MMP-2, collagen-I, collagen-III and masson trichrome

Groups					
	**Control**	**Diabetic Control**	**Diabetic Hypertensive Control**	**Rosiglitazone**	**Rosiglitazone + Felodipine**

TGs (mg/dL)	79.50 ± 2.65	95.45 ± 6.38^	98.95 ± 7.29	75.15 ± 4.94#	75.40 ± 7.31#

T-Chol (mg/dL)	133.38 ± 7.17	194.59 ± 14.36^	227.76 ± 19.94*	158.90 ± 6.82#	151.98 ± 6.83#a

LDL-C (mg/dL)	72.86 ± 9.39	140.22 ± 14.91^	175.13 ± 19.69*	104.15 ± 7.64#	96.01 ± 8.08#a

HDL-C (mg/dL)	44.62 ± 3.62	35.28 ± 2.22^	32.84 ± 1.62*	39.72 ± 2.47#	40.89 ± 2.77#

TNF-α (ng/mL)	203.19 ± 10.88	249.10 ± 13.73^	274.58 ± 17.38*	217.45 ± 18.23#	211.93 ± 17.91#

TGF-β	1.06 ± 0.09	2.01 ± 0.23^	2.17 ± 0.29	1.16 ± 0.10#	1.10 ± 0.07#

MMP-2	0.82 ± 0.09	0.06 ± 0.04^	0.11 ± 0.05*	0.41 ± 0.12#	0.43 ± 0.22#

Collagen-I	0.11 ± 0.04	0.80 ± 0.21^	1.06 ± 0.27*	0.55 ± 0.09#	0.38 ± 0.11#a

Collagen-III	0.06 ± 0.02	0.11 ± 0.03^	0.13 ± 0.06	0.06 ± 0.02#	0.06 ± 0.02#

Masson trichrome	13.54 ± 1.31	10.79 ± 1.86^	22.34 ± 6.29*	8.79 ± 1.44#	10.45 ± 2.96#

Values are expressed as mean ± SD. Number of rats per group n = 10.					

STZ, streptozotocin; TGs, triglycerides; T-Chol, total cholesterol; LDL-C, low density lipoprotein cholesterol; HDL-C, high density lipoprotein cholesterol; TNF-α, tumor necrosis factor alpha; TGF-β, transforming growth factor beta; MMP-2, matrix metalloproteinase-2

^ Significant difference from normal control at P ≤ 0.05.

* Significant difference from diabetic control at P ≤ 0.05.

# Significant difference from diabetic hypertensive control at P ≤ 0.05.

a Means of treated groups with different superscript are significantly different from each other at P ≤ 0.05.

DC animals showed a significant increase in their T-Chol levels by 45.89% in comparison with group C. Furthermore, DHC animals showed also a significant increase in their T-Chol levels by 17.05% in comparison with DC animals group. At the other side, there was a significant decrease between group R in comparison to DHC group in their T-Chol levels by 30.23%. Also there was a significant decrease between the group R+F in their T-Chol levels by 33.27% in comparison with DHC data (Table 2).

HDL-C levels decreased significantly in DC animals in comparison with group C by a level of 20.93%. Also, there was a significant decrease between DHC data in their HDL-C levels by 6.92% in comparison with DC data. Meanwhile, there was nearly the same significant increase in group R and the group R+F levels by (20.95% and 24.51% respectively) in comparison with DHC data (Table 2).

LDL-C levels showed a significant increase in DC animals by 92.45% in comparison with group C. Also, there was a significant increase in DHC animals in their LDL-C levels by 24.9% in comparison with DC data. On the other hand, LDL-C levels decreased significantly in group R in comparison with DHC group by a level of 40.53%. Moreover, LDL-C levels decreased significantly in the group R+F in comparison with DHC group by a level of 45.18% (Table 2).

**Figure 1:**
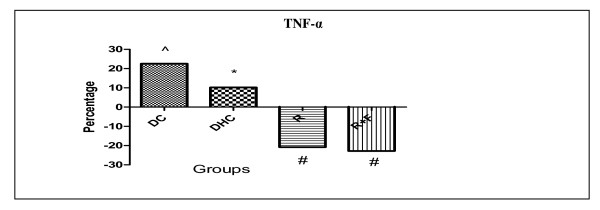
**Percentage change of TNF-α in group DC; group DHC; group R and group R+F for one month**. Each value is expressed in ng/mL. (^) Significant difference from normal control at P < 0.05. (*) Significant difference from diabetic control at P < 0.05. (#) Significant difference from diabetic hypertensive control at P < 0.05. Using one way ANOVA followed by Tukey test as post hoc test.

There is a significant increase in TNF-α level in DC group by 22.59% in comparison with group C. Furthermore, DHC animals showed also significant rise in their TNF-α levels by 10.23% in comparison with DC animals group. However, group R and group R+F induced nearly the same significant decrease in their TNF-α level by (20.81% and 22.82% respectively) in comparison with DHC data (Table 2, Figure 1).

**Figure 2:**
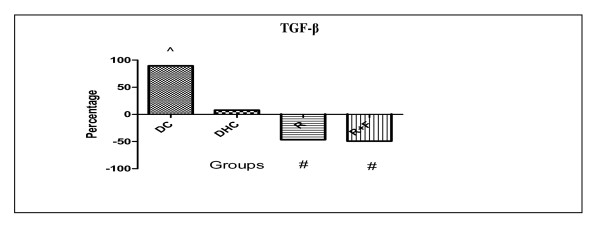
**Percentage change of TGF-β in group DC; group DHC; group R and group R+F for one month**. (^) Significant difference from normal control at P < 0.05. (#) Significant difference from diabetic hypertensive control at P < 0.05. Using one way ANOVA followed by Tukey test as post hoc test.

TGF-β levels showed a significant rise in DC group by 89.62% in comparison with group C. But, DHC group showed no significance in their TGF-β levels in comparison with DC group. However, group R and group R+F induced nearly the same significant decrease in their TGF-β levels by (46.54% and 49.31% respectively) in comparison with DHC group data (Table 2, Figure 2).

**Figure 3:**
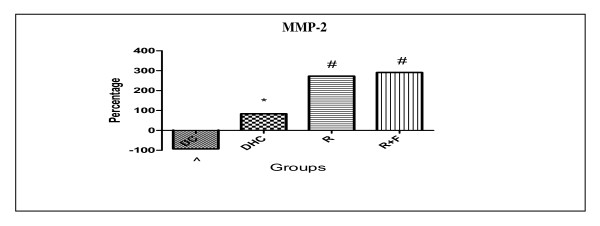
**Percentage change of MMP-2 in group DC; group DHC; group R and group R+F for one month**. (^) Significant difference from normal control at P < 0.05. (*) Significant difference from diabetic control at P < 0.05. (#) Significant difference from diabetic hypertensive control at P < 0.05. Using one way ANOVA followed by Tukey test as post hoc test.

MMP-2 levels DC showed a significant decrease in DC group by 92.68% in comparison with group C. But, DHC group showed a significant rise in their MMP-2 levels by 83.33% in comparison with DC group. However, group R and group R+F data induced nearly the same significant rise in their MMP-2 levels by (272.73% and 290.91% respectively) in comparison with DHC group data (Table 2, Figure 3).

Our results revealed that, there is no significant difference between TGF-β and MMP-2

**Figure 4:**
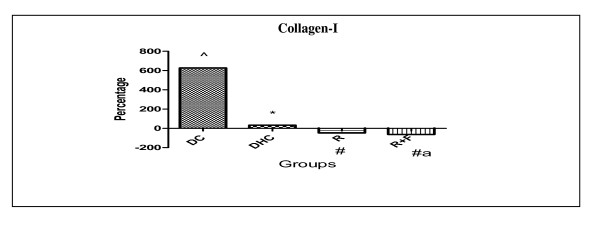
**Percentage change of collagen-I in group DC; group DHC; group R and group R+F for one month**. (^) Significant difference from normal control at P < 0.05. (*) Significant difference from diabetic control at P < 0.05. (#) Significant difference from diabetic hypertensive control at P < 0.05. a....., Means of treated groups with different superscript are significantly different from each other at P < 0.05. Using one way ANOVA followed by Tukey test as post hoc test.

There was a significant rise in their collagen I levels in DC group by 627.27% in comparison with group C. Furthermore, DHC animals showed also significant rise in their collagen I levels by 32.50% in comparison with DC animals group. However, group R induced a significant decrease in their collagen I levels by 48.11% in comparison with DHC data group. Also, group R+F induced a significant decrease in their collagen I levels by 64.15% in comparison with DHC data group (Table 2, Figure 4).

**Figure 5:**
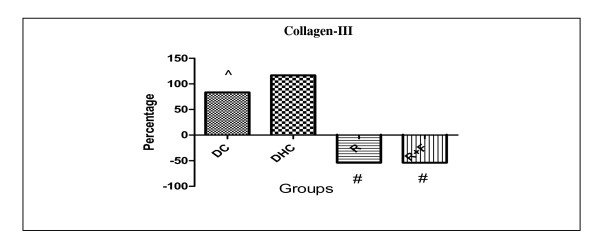
**Percentage change of collagen-III in group DC; group DHC; group R and group R+F for one month**. (^) Significant difference from normal control at P < 0.05. (*) Significant difference from diabetic control at P < 0.05. (#) Significant difference from diabetic hypertensive control at P < 0.05. Using one way ANOVA followed by Tukey test as post hoc test.

There was a significant rise in their collagen III levels in DC group by 83.33% in comparison with group C. But, DHC group showed no significance in their collagen III levels in comparison with DC animals group. However, group R and group R+F induced the same significant decrease in their collagen III levels by nearly 53.85% in comparison with DHC data group (Table 2, Figure 5).

Light microscope examination of H&E stained sections of the cardiac muscle of the group C rats revealed that the cardiac muscle fibers are connected end to end by intercalated discs appeared with central and oval vascular nuclei.

H&E stained sections of the cardiac muscle of the DC group rats showed degeneration in some of the cardiac muscle fibers in the form of separation and fragmentation of the cardiac muscle fibers.

Examination of H&E stained sections of the cardiac muscle of the DHC group rats showed longitudinal section in the cardiac muscle fibers showing hypertrophy of the cardiomyocytes.

The cardiac muscle fibers of rats receiving rosiglitazone treatment alone showed mild fragmentation and degeneration of the cardiomyocytes in H&E stained sections.

Histological examination of H&E stained sections of the cardiac muscle fibers of rats receiving combined treatment of rosiglitazone and felodipine revealed a nearly normal appearance of the cardiac muscle fibers with no fosi of degeneration.

**Figure 6:**
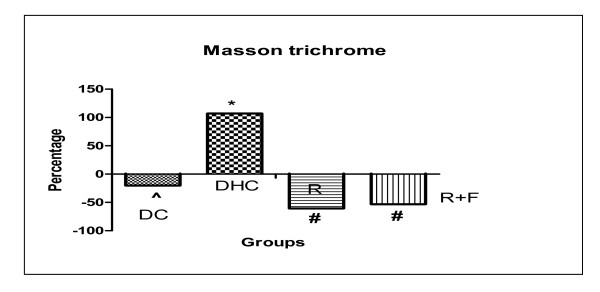
**Percentage change of masson trichrome in group DC; group DHC; group R and group R+F for one month**. (^) Significant difference from normal control at P < 0.05. (*) Significant difference from diabetic control at P < 0.05. (#) Significant difference from diabetic hypertensive control at P < 0.05. Using one way ANOVA followed by Tukey test as post hoc test.

Our results illustrated that, there was a significant decrease in their area percentage of masson trichrome stained connective tissue (CT) fibers of cardiac muscle levels in DC group by 20.285% in comparison with group C. Meanwhile, DHC rats showed a significant increase in their area percentage of masson trichrome stained CT fibers levels by 106.975% in comparison with DC rats group. However, group R induced a significant decrease in their area percentage of masson trichrome stained CT fibers levels by 60.67% in comparison with DHC data group. Also, group R+F induced a significant decrease in their area percentage of masson trichrome stained CT fibers levels by 53.24% in comparison with DHC data group. Moreover, there is no significant difference between R group and R+F group rats (Table 2, Figure 6).

## Discussion

Diabetes is a growing global health problem with type 2 DM being now an epidemic [55]. The major clinical consequences of type 2 DM are mortality and morbidity from vascular complications [56]. Moreover patients with type 2 DM have an increased risk of premature development and accelerated progression of atherosclerosis [57].

The pathogenesis of CVD in DM is multi-factorial and can be affected by many factors such as hyperglycemia, increased production of fatty acids, and altered lipoproteins [58]. Recently, important factors were proposed to initiate CV complications in type 2 DM, including cardiac fibrosis and inflammation [59]. Consequently, there have been considerable recent efforts to focus on non-traditional risk factors for CV events in type 2 DM patients' population.

Streptozotocin (STZ) is a synthetic antineoplastic agent that is classifically an anti-tumor antibiotic and chemically is related to other nitrosureas used in cancer chemotherapy. STZ injury, induced diabetes can begin an autoimmune process that results in destruction of the Langerhans islets beta cells and toxicity of beta cells of pancreas with emergence of clinical diabetes within 2-4 days [60].

The result of our study explained that, in STZ- induced diabetic and DH groups when compared to the normal control healthy group, there was a significant increase in BG. This result was consistent with many studies [60-63].

Our study explained that, there was a significant increase in BG levels between DC and DHC rats (group II and III, respectively) compared with group C. Also this is supported by a significant positive correlation between BG and BP. This study is confirmed with the study of [64].

In the contrary, rosiglitazone showed a significant decrease in BG levels in treated groups (groups R and R+F, respectively). But there is no significantly decrease in group R+F which is treated with rosiglitazone and felodipine combination in comparison to group R which is treated with rosiglitazone only. This study is consistent with [62].

From these data, it is clear that rosiglitazone which acts as peroxisome proliferator activated receptor gamma had the greatest role in treatment of DM. But at the other hand, there was no role for felodipine in treatment of DM, so there is no significant decrease in BG when felodipine was added to treatment.

Hypertension is a major risk factor for CHD and can induce several other risk factors [65]. Hence, regarding the blood pressure; BP was elevated in the DC and DHC rats (groups II and III, respectively) compared with group C. This was also the case in the studies conducted on hypertensive type 2 DM patients [66]. Furthermore, this is supported by a positive correlation between BG and BP.

In the contrary, there is a significant decrease in group V which is treated with rosiglitazone and felodipine combination due to felodipine effect in comparison to DHC rats (group III). But on the other hand, there is no significant decrease in group R which is treated with rosiglitazone only in comparison to DHC rats (group III).

From these data, it is obvious that treatment combination of rosiglitazone and felodipine had the upper hand in decreasing BP over rosiglitazone treatment only because of the role of felodipine which acts as calcium channel blocker [67].

It is reported that prolonged exposure to hyperglycemia is recognized as the primary casual factor in the pathogenesis of diabetic cardiac complications. Even in the upper limits of normal range and independent of other known risk factors, hypertension could be predictor of coronary events [68,69]. These findings confirm that hyperglycemia induces a large number of alterations in the cardiac tissue that potentially promote increasing of interstitial fibrosis and myocyte apoptosis [70,71]. Several studies have attempted to find the link between hyperglycemia and development of coronary atherosclerosis [72,73]. Therefore, the highest BG and BP values were observed in DHC group.

We should be aware of the important relationship between type 2 DM and cardiac fibrosis.

Hence, the present study was conducted aiming to evaluate the role of lipid profile, TNF- α, TGF-β, Collagen I, Collagen III and MMP-2 in type 2 DM hypertensive patients who developed cardiac fibrosis. Correlating these parameters in the DHC groups, as well as their relation with glycemic status was also a target in this study. In order to achieve our aim, we assessed the levels of these biochemical markers in DHC rats with and without cardiac fibrosis and compared them with group C. This was done in an attempt to explore the possible relationships between hyperglycemia, hypertension, dyslipidemia and inflammation as well as cardiac fibrosis.

It was stated that dyslipidemia is a recognized risk factor for CVD in DM. In our study, all diabetic rats showed significant dyslipidemia compared with the healthy control subjects. It was found that the levels of TAG, T-Chol, and LDL-C were significantly increased in DC and DHC groups (II and III, respectively) as compared to the group C. On the other hand, the level of HDL-C was significantly reduced in both groups, as compared to the group C. Furthermore, this fact is supported with a significant positive correlation between BG and T- Chol [74].

In the contrary, there was a significant increase for HDL-C in group R which was treated with rosiglitazone only and group R+F which was treated with combination of rosiglitazone and felodipine in comparison to DHC rats. But there was no significant difference between group R+F and group R. It is obvious that there is no great role for felodipine in increasing HDL-C for DH rats.

Furthermore, there was a significant increase for T-Chol in DC and DHC groups (II and III, respectively).

But, there was a significant decrease for T-Chol in group R which was treated with rosiglitazone only and group R+F which was treated with combination of rosiglitazone and felodipine in comparison to DH rats. And also, there is a significant decrease for T-Chol in group R and R+F.

The work was reported that; when raised TAG co-exists with an atherogenic T-Chol profile, the overall risk of atherosclerosis is enhanced. The more features of metabolic syndrome; a rat has the greater the risk which is made much worse by concomitant LDL-C elevation [75,76].

Hyperglycemia may be associated with proatherogenic modifications to plasma lipoproteins [77]. TNF-α is one of the key inflammatory mediators expressed during a variety of inflammatory conditions, and takes part in a variety of physiological and pathological phenomena as increasing of its expression in coronary arteries [78].

In our study, there was a significant increase in DC and DHC rats groups (II and III, respectively) as compared with group C. Also there was a significant decrease for TNF-α in group R which was treated with rosiglitazone only and in group R+F which was treated with combination of rosiglitazone and felodipine in comparison to DHC rats. Furthermore there was no significant difference between group R and R+F.

These findings confirm that felodipine has no any role in improvement of TNF-α expression in cardiac fibrosis cases. So it is obvious that there is a potent relation between increasing of TNF-α expression and (CADs) [79]. Also, this is supported with a significant positive correlation between BG and TNF-alpha.

The myocardium is composed of cardiac myocytes enveloped in a dense extracellular network of collagen, the main structural protein. Cardiac myocytes account for 70% to 75% of the myocardium by cell volume but only 25% to 30% by cell number [80].

Cardiac fibroblasts are the most abundant cell type of the myocardium and produce various proteins found in the extracellular matrix (ECM), including the major fibrillar collagens type I and III, which comprise the bulk of the ECM, as well as collagenases, fibronectin, and vitronectin [81].

Our study explained that, there was a significant increase for collagen I in DC and DHC rats groups (II and III, respectively) in comparison to group C (group I).

On the other hand, there was a significant decrease in group R which was treated with rosiglitazone only and group R+F which was treated with rosiglitazone and felodipine combination in comparison to DHC rats (group III).

Furthermore, there was a significant decrease within groups (R and R+F), this elucidates that group R+F which was treated with rosiglitazone and felodipine combination is more potent action on collagen I than group R which was treated with rosiglitazone only.

These findings confirm that the treatment with combination of rosiglitazone and felodipine has the upper hand on treatment with rosiglitazone alone which clarify that, this combination has a great role on attenuating cardiac perivascular fibrosis due to the presence of felodipine which acts as calcium channel blocker due to the blood pressure regulation. And also, there was a significant increase for collagen III in DC and DHC rats in groups (II and III, respectively) in comparison to group C (group I). But there was no significant difference within groups (DC and DHC). But, there was a significant decrease in group R which was treated with rosiglitazone only and in group R+F which was treated with rosiglitazone and felodipine combination when compared with DHC rats (group III). It is obvious that, there was no significantly difference between group R and group R+F. It is confirmed that there was no correlation between blood glucose and collagen III, but there was a positive correlation between BP and collagen III. So it is cleared that the addition of felodipine to treatment has no effect on improvement of collagen III. This result is opposed with the study of [40].

TGF-β is a multifunctional cytokine that acts on a wide assortment of cell types regulating both cell growth and differentiation. TGF-β can have potent inhibitory effects on T-cell proliferation, cytokine production, and cytolytic functions [82].

Our study showed a significant increase of TGF-β in DC and DHC rats in groups (II and III, respectively) in comparison to group C (group I). But, there was no significantly difference between groups (DC and DHC).

On the other hand, there was a significant decrease in group R which was treated with rosiglitazone only and group R+F which was treated with rosiglitazone and felodipine combination in comparison to DHC rats (group III). But, at the same time, there was no significantly difference between groups (R and R+F).

It is clear that, the results of TGF-β clarified that treated groups even with rosiglitazone only (group R) or with rosiglitazone and felodipine combination (group R+F) are nearly near to group C (group I). The conclusion for TGF-β shows that it is a very good indicator for determination of cardiac fibrosis as TGF-β_1 _is produced normally at low levels in cardiac tissue. However, when myocardial injury occurs, TGF-β_1 _is released in large quantities by injured myocytes, fibroblasts, infiltrating macrophages and lymphocytes. [83] is consistent with our study.

MMPs are an endogenous family of proteolytic enzymes that play an important role in extracellular matrix (ECM) turnover and cardiac remodeling. The MMP family consists to date of more than 20 species including collagenases (such as MMP-1 and MMP-13), gelatinases (such as MMP-2 and MMP-9), stromelysin (MMP-3) and membranous type MMP (such as MT1-MMP). MMP-2 is produced by cardiomyocytes, cardiac fibroblasts and endocardial cells. Alterations in the expression and activity of MMP-2 have been demonstrated in a number of pathophysiological conditions. As increased MMP-2 expression should result in a decreased level of cardiac interstitial collagen, in which large increases in gelatinase activity were associated with increased total collagen content. In the setting of myocardial ischemia/reperfusion, acutely elevated MMP-2 activity contributes to myocardial stunning in human and rat hearts, independent of an effect on the ECM. During post-ischemic reperfusion, activation of intracellular MMP-2 leads to cleavage of the contractile regulatory protein troponin-I. Conversely, myocardial stunning was attenuated by inhibition of MMP-2 activity [37,84-86].

In our study, low MMP-2 level was clearly observed in type 2 DM and DH rats, which is consistent with the study of [85] who stated that MMP-2 is a good indicator for evaluating cardiac fibrosis in degenerative diseases like DM. The decreased MMP-2 significant level observed in our DC and DHC rat groups (II and III, respectively) when compared to group C.

But, on the other hand, there was a significant increase in group R which was treated with rosiglitazone only and in group R+F which was treated with rosiglitazone and felodipine combination in comparison to DHC rats group. Also, at the same time, there was no significant difference between groups R and R+F. This is supported by a positive correlation between BG and MMP-2. [87] is also consistent with this study.

It is obvious that, there is no significant difference between TGF-β and MMP-2.

In our study, the area percentage of masson trichrome stained CT fibers of treated groups were nearly the same as control healthy group that have excellent results than that of DHC group rats, which emphasize the great role of rosiglitazone and felodipine treatment in attenuating the cardiac fibrosis.

## Conclusion

Our study revealed that DH rats develop hyperglycemia, hypertension and cardiac fibrosis. They also resulted in metabolic dyslipidemia, increased TNF-α, TGF-β, collagen-I and collagen-III and decreased MMP-2. Our results demonstrate that treatment with rosiglitazone alone is able to improve the hyperglycemia, dyslipidemia, and to decrease TNF-α, TGF-β, collagen-I and collagen-III and increased MMP-2 but within a minimal effect. Meanwhile, it had no effect on attenuating the hypertension which may lead to CADs again. Moreover, the combined treatment with rosiglitazone and felodipine is able to improve the hyperglycemia and has a great effect on attenuating the hypertension. The combined treatment is also able to improve dyslipidemia, and to decrease TNF-α, TGF-β, collagen-I and collagen-III and increased MMP-2 but within a greater effect than treatment with rosiglitazone alone.

## Competing interests

The authors declare that they have no competing interests.

## Authors' contributions

HEM put the idea of research and developed the study protocol, supervised samples and results interpretation and correlation assessments, and contributed to the revising of the manuscript. MEA participated in protocol writing, as well as experimental work, helped in statistical analysis, supervised samples' collection, preparation and analysis, provided superb scientific guidance regarding interpretation and presentation of results, and helped to draft the manuscript. MMH carried the designed protocol, performed the statistical analysis and contributed to the writing of the manuscript. All authors read and approved the final manuscript.
